# Combining automated peak tracking in SAR by NMR with structure-based backbone assignment from ^15^N-NOESY

**DOI:** 10.1186/1471-2105-13-S3-S4

**Published:** 2012-03-21

**Authors:** Richard Jang, Xin Gao, Ming Li

**Affiliations:** 1 David R Cheriton School of Computer Science, University of Waterloo, Waterloo, Ontario, N2L 3G1, Canada; 2 Division of Mathematical and Computer Sciences and Engineering, King Abdullah University of Science and Technology, Thuwal, 23955, KSA

## Abstract

**Background:**

Chemical shift mapping is an important technique in NMR-based drug screening for identifying the atoms of a target protein that potentially bind to a drug molecule upon the molecule's introduction in increasing concentrations. The goal is to obtain a mapping of peaks with known residue assignment from the reference spectrum of the unbound protein to peaks with unknown assignment in the target spectrum of the bound protein. Although a series of perturbed spectra help to trace a path from reference peaks to target peaks, a one-to-one mapping generally is not possible, especially for large proteins, due to errors, such as noise peaks, missing peaks, missing but then reappearing, overlapped, and new peaks not associated with any peaks in the reference. Due to these difficulties, the mapping is typically done manually or semi-automatically, which is not efficient for high-throughput drug screening.

**Results:**

We present PeakWalker, a novel peak walking algorithm for fast-exchange systems that models the errors explicitly and performs many-to-one mapping. On the proteins: hBcl_XL_, UbcH5B, and histone H1, it achieves an average accuracy of over 95% with less than 1.5 residues predicted per target peak. Given these mappings as input, we present PeakAssigner, a novel combined structure-based backbone resonance and NOE assignment algorithm that uses just ^15^N-NOESY, while avoiding TOCSY experiments and ^13^C-labeling, to resolve the ambiguities for a one-to-one mapping. On the three proteins, it achieves an average accuracy of 94% or better.

**Conclusions:**

Our mathematical programming approach for modeling chemical shift mapping as a graph problem, while modeling the errors directly, is potentially a time- and cost-effective first step for high-throughput drug screening based on limited NMR data and homologous 3D structures.

## Background

X-ray crystallography and NMR spectroscopy are the predominant methods for experimental 3D protein structure determination. The advantage of NMR over any other method is that the protein sample can be studied at atomic resolution in solution, and in special cases even in living cells (in-cell NMR) [1,2]. In addition to structure determination, NMR has been used successfully in protein-protein interaction studies [3], studies on protein dynamics [4], and in drug design and screening [5]. Among the more successful NMR methods for drug design and screening, fragment-based methods, such as SAR by NMR [6,7], have found their way in pharmaceutical companies and have resulted in discoveries that are currently undergoing clinical trials [8]. In SAR by NMR and other NMR studies, chemical shift mapping is used to identify the atoms in a target protein that experience chemical shift changes upon introduction of a ligand or upon changes in environmental conditions.

*The chemical shift*, *δ*, of an atom is its resonance frequency (in units of ppm) measured by NMR experiments. We consider the chemical shifts of three NMR-active isotopes with focus on the latter two: ^13^C, ^15^N and ^1^H. Among the large variety of NMR spectra, only 2D HSQC, 3D NOESY, and 3D TOCSY will be discussed. Each 2D HSQC peak gives the chemical shifts of an N, H*^N ^*group, including backbone amides and side chains with amide groups. Our focus is on the backbone amide chemical shifts, which serves as an identifier for an amino acid residue. Each 3D NOESY peak (NOE) consists of three chemical shifts: N, H*^N ^*of an amide group, and another proton that is within a distance of about 5Å from the H*^N^*. Therefore, each NOE corresponds to a H*^N^*- H contact. Each 3D TOCSY peak consists of the chemical shifts of an amide group, and a proton within the same amino acid as the amide. Therefore, TOCSY gives the side chain protons. In this work, we consider only H*^α ^*and H*^N^*.

**Figure 1:**
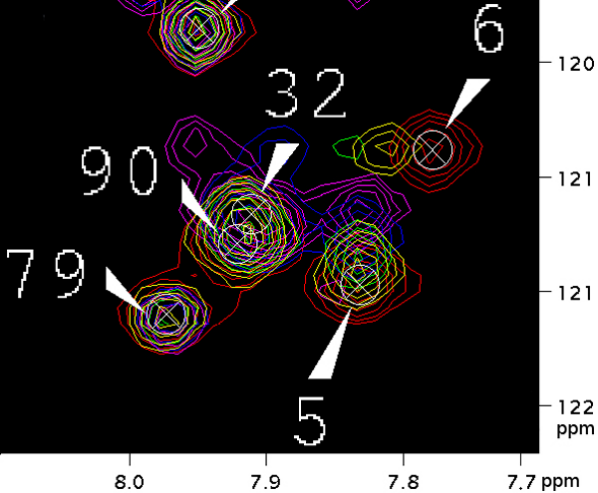
**A region of an overlay of five ^15^N-HSQC spectra at increasing ligand concentrations.** Each peak is represented by its contours. Red peaks correspond to the unbound protein; yellow to the protein at 1:8 saturation; green to 1:4; blue to 1:2; and magenta to the fully saturated protein. The maxima of the red peaks are labeled by crosshairs and residue numbers. The ligand is unlabeled, so its peaks are not present.

Figure 1 shows a small region of an overlay of five ^15^N-HSQC spectra of a protein titrated at increasing ligand concentrations. Each "peak" can be picked manually or with an automated peak picking tool [9]. Normally, the assignment of peak to amino acid residue, known as the resonance assignment, is known for the peaks of the unbound protein. The NMR spectrum with known resonance assignment shall be referred to as the reference spectrum, while the other spectra shall be the perturbed spectra. The perturbed spectrum of the fully saturated protein shall be referred to as the target spectrum. In chemical shift mapping, the goal is to trace a path from target peaks to reference peaks, or vice versa, to obtain a resonance assignment for the target peaks. From the figure, we can see that residue 6 has moved, and the mappings for 32 and 90 are ambiguous due to peak overlap. The "peak walking" pattern observed in the figure applies to fast exchange systems, which is the focus of this paper. Many experimental schemes for studying ligand binding are for fast exchange systems [10]. After the assignment has been determined, one can compute binding constants and rate of change parameters, such as by using Auto-FACE [11].

Typically, chemical shift mapping is done manually or semi-automatically due to errors, noise, peak overlap, and missing data. This manual work can be tedius and time consuming if the protein is large, if there are many spectra, or if there are many ambiguous mappings. Moreover, results derived manually is naturally biased, so the results can be difficult for others to reproduce. To our knowledge, there are only a few automated methods for this problem, and they all produce one-to-one mappings rather than allowing for ambiguity. Nevertheless, automated methods are necessary for high-throughput drug screening.

FELIX-Autoscreen [12] formulates the assignment of peaks in the reference spectrum to peaks in a perturbed spectrum as a bipartite graph matching problem, such that the sum of the chemical shift and peak shape differences is minimized. Their approach of optimizing the sum of the distances is better than choosing the peak nearest to each reference peak because the local greedy approach disregards the mappings of other peaks nearby, which results in errors. Dummy peaks were used to handle missing data, and peaks were picked on the fly during the execution of their algorithm. To handle more than one set of perturbed spectra, the bipartite matching algorithm was repeated successively, where the current perturbed spectrum becomes the new reference spectrum once it has been mapped. They tested their approach on a 74-residue protein domain in 8 different ligand concentrations, and obtained results similar to their manual efforts. The successive approach, however, is a local greedy approach that does not consider all the spectra simultaneously, so information about potential peak movements in later perturbed spectra are ignored.

NvMap [13] also used a greedy algorithm to successively match perturbed spectra. However, unlike FELIX-Autoscreen, when matching the reference to a perturbed spectrum, the sum of the distances was not used. Instead, the pair of reference and perturbed peaks with the shortest distance was chosen and removed from consideration, and then the process was repeated for the next shortest. They tested their method on 97 residues of the SUMO protein on 2 different ligands, each at 6 different ligand concentrations. They obtained an average accuracy of 95%. The main source of error was overlapping peaks within a spectrum, where only one of the peaks was picked and added to the peak list. An older method, MUNIN [14], identifies spectra similarity, but not peak paths. By examining a specific subregion in a mixture of different spectra, where only one had binding, it was able to identify the spectrum with binding present.

For large proteins, ambiguous mappings are inevitable. Rather than finding the unique mapping between peaks in the target to peaks in the reference, we find a set of plausible reference peaks for each target peak, where plausibility is determined by a scoring function. If the residue assignment for the reference is known, then the mappings give a set of possible residues for each target peak; e.g., ILE 3, LEU 27, LEU 78. We want this set to be small, but yet contain the correct amino acid. In this paper, we present a novel peak walking model that describes the movements that peaks can make, and an approach that generates high scoring mappings by enumerating high scoring paths based on this model. Unlike previous methods, errors are modeled explicitly without using dummy peaks. We call our method PeakWalker. We tested PeakWalker on 3 proteins with publicly available peak lists: UbcH5B titrated with Not4 [15]; hBcl_XL _with BH3I-1 [11,16]; and histone H1 at 2 different temperatures [17]. At 218 residues minus a removed flexible loop region R45 to A84, which was removed from the DNA sequence prior to NMR, hBcl_XL _is much larger than the proteins tested by other automated methods. The average accuracy on the test set was at least 96%, with an average of less than 1.5 amino acids predicted per target peak. We compare PeakWalker to a greedy approach similar to that used by NvMap, but modified to return multiple mappings. We also tested PeakWalker by varying the number of noise peaks.

In the second half of this paper, we describe our structure-based resonance assignment method, PeakAssigner, which takes the output of PeakWalker as input, and then resolves the mapping ambiguities using 3D ^15^N-NOESY and the 3D structure of a homologous protein. In chemical shift perturbation studies, a 3D structure is often available, such as from the Protein Data Bank (PDB) [18]. It is often the case that the bound structure of the protein is similar across different ligands that can bind to it, so that one bound structure can be used for studying different ligands. Therefore, structure-based resonance assignment methods [19-24] are ideal for disambiguating the mappings. Currently, there are no automated backbone resonance assignment methods that use only a series of ^15^N-HSQC spectra and ambiguous NOEs from ^15^N-NOESY spectra. NOEnet [20] requires unambiguous NOEs, such as from 4D NOESY. The Nuclear Vector Replacement (NVR) [21,23] approach requires a sparse set of unambiguous NOEs from 3D NOESY, residual dipolar couplings (RDC), and amide exchange rates. The contact replacement (CR) [22] method can handle ambiguous NOEs from 3D ^15^N-NOESY, but it also requires 3D ^15^N-TOCSY, and 3D HNHA.

Our previous work on structure-based resonance assignment [19] has requirements similar to the CR method except that instead of HNHA, it requires a known resonance assignment from a protein mutant, which serves as a reference. We were able to perform a fully automatic backbone resonance assignment from automatically picked peaks for a small protein. However, using 3D ^15^N-TOCSY and a similar resonance assignment limited the practicality of the method. In large proteins, the TOCSY can have many overlapped peaks or many missing peaks if the protein is deuterated. In addition, each reference peak can have many corresponding target peaks, so there can be many ambiguous mappings.

In this work, we no longer use TOCSY. The TOCSY was previously used to identify possible amino acid types for each target peak, and this was used to reduce the number of ambiguous mappings. To reduce ambiguity without TOCSY, a series of perturbed spectra could be used. The TOCSY was also used to obtain the chemical shifts of the H*^α ^*atoms for matching against NOESY peaks. Such H*^α ^*chemical shifts are available in the NOESY spectrum, but in a more noisy form. We have also added a further improvement. The constraint that each NOESY peak is assigned to at most one contact was not enforced in our previous algorithm. In adding this constraint, our new algorithm not only performs resonance assignment, but also backbone NOE assignment and H*^α ^*assignment, simultaneously. Although NOE and H*^α ^*assignment is not the main output of our algorithm, we show that by performing them, there is an improvement in resonance assignment accuracy, on average. This is demonstrated with simulated NOESY peaks from the protein structures [PDB:1KA5], [PDB:1EGO], [PDB:1G6J], [PDB:1SGO], and [PDB:1YYC]. On hBcl_XL_, UbcH5B, and histone H1, PeakAssigner achieves an average accuracy of over 94%.

At the end of this paper, we briefly consider the slow-exchange case. In *slow exchange*, the peaks for both the free and bound state may appear in the spectra at the same time, with the intensity of the peak signals proportional to the concentration of each state. If the protein in Figure 1 undergoes slow exchange, only the red and magenta peaks would be present. In the unbound protein, only the red peaks are present. As the ligand concentration increases, for residues undergoing chemical exchange, magenta peaks will appear at increasing peak intensities relative to the corresponding red peaks, which disappear in the fully saturated case.

## Results and discussion

This section will describe the mathematical model used by PeakWalker and PeakAssigner, followed by the test results. The test data is described in detail in the Methods.

### Peak walking problem

PeakWalker is based on k-dimensional maximum matching, which is NP-Complete and APX-complete for *k *> 2 [25,26]. For *k *= 2, the problem is maximum bipartite matching, which is solvable in polynomial time [27]. Consider the peak lists in increasing ligand concentrations {*T_i _*| *i *∈ [0, 1, ..., *k *- 1]}. *T*_0 _denotes the reference peaks, and *T*_*k*-1 _denotes the target peaks. Each peak is represented by a vertex. The chemical shift change or distance is used to draw edges between vertices. The distances used in this work include

(1) $$\begin{array}{llll} \Delta \delta_{N}\left(h,h^{\prime }\right) & =\;|\delta_{N}\left(h\right)-\delta_{N}\left(h^{\prime }\right)|\; & & \;\\ \Delta \delta_{H^{N}}\left(h,h^{\prime }\right) & =\;|\delta_{H^{N}}\left(h\right)-\delta_{H^{N}}\left(h^{\prime }\right)|\; & & \;\\ \Delta \delta_{NH}\left(h,h^{\prime }\right) & =\Delta \delta_{N}\left(h,h^{\prime }\right)+10\times \Delta \delta_{H^{N}}\left(h,h^{\prime }\right)\; & & \;\\ & \; & \end{array}$$

where *δ_N_*(*h*) is the function that returns the N chemical shift of *h*, $\delta_{H^{N}}\left(h\right)$ the H*^N ^*chemical shift of *h*, and the 10 comes from the gyromagnetic ratio of ^1^H and ^15^*N*. Euclidean distance and various types of weightings can also be used to measure chemical shift change [28]. For peaks *h *∈ *T_i _*and *h*' ∈ *T*_*i*+1_, an edge is drawn between them if Δ*δ_N_*(*h*, *h*') ≤ *t_N _*and $\Delta \delta_{H^{N}}\left(h,h^{\prime }\right)\leq t_{H^{N}}$, where *t_N _*and $t_{H^{N}}$ are user-specified thresholds. For UbcH5B and histone H1, 1.0 ppm and 0.2 ppm were used for *t_N _*and $t_{H^{N}}$, respectively. This is comparable to the thresholds used by FELIX-Autoscreen [12]. Smaller thresholds of 0.75 ppm and 0.125 ppm were used for hBcl_XL _because it has more perturbed spectra, so the chemical shift changes are expected to be more gradual. Edges are not drawn between vertices within the same peak list, so the *T_i_*'s are disjoint.

**Definition 1**. *The *maximum weighted k-dimensional matching *on instance T *⊆ *T*_0 _× *T*_1_× ... × *T*_*k*-1_, *where the T_i_'s are disjoint, is the set of paths M *⊆ *T that maximizes some scoring function on M subject to the constraint that for any pair of paths x*, *y *∈ *M*, *x and y have no vertices in common*.

**Figure 2:**
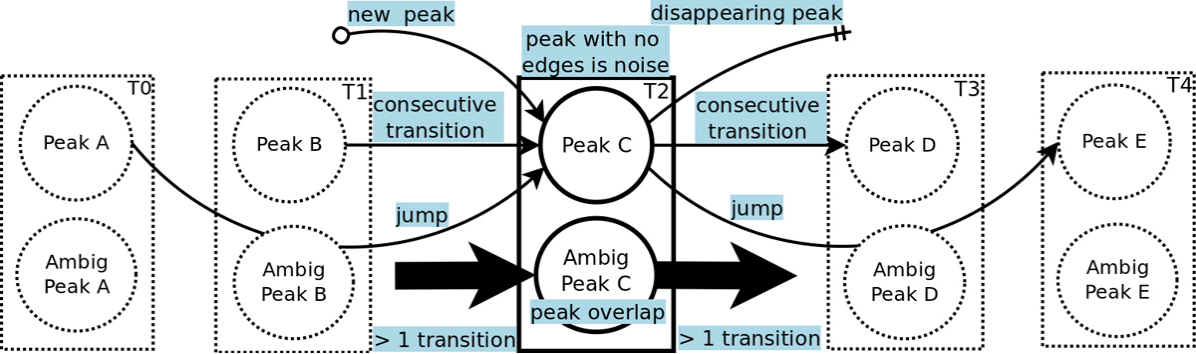
**Peak walking model for fast exchange.** The allowable transitions include new peak, consecutive, jump, and disappearing. A peak is either ambiguous or unambiguous. An ambiguous peak can have multiple transitions, whereas an unambiguous peak can have only one in- and one out-transition.

The problem is equivalent to finding the best scoring set of vertex-independent paths from reference peaks to target peaks. Our problem is a constrained version of this problem, where the allowable paths are limited by the peak movements defined by a peak walking model. Figure 2 illustrates the model. A peak in *T_i _*can transition to nearby peaks in *T*_*i*+1 _within *t_N _*and. $t_{H^{N}}$ These transitions shall be referred to as consecutive transitions. A peak can also disappear permanently, or disappear in *T*_*i*+1_, but then reappear in *T*_*i*+2_. The former shall be referred to as a disappearing transition, and the latter a jump. Only jumps of length 2 are explicitly modeled. Finally, a peak in *T_i _*may correspond to a residue with no peaks in *T_j_*, ∀*_j _*<*i*. These shall be referred to as new peaks. Transitions correspond to directed edges in the graph. New peaks have no predecessor peak, and disappearing peaks have no successsor. Both of these peaks result in subpaths. Peaks that have almost identical chemical shifts may have only one peak present in the peak list due to peak overlap. To handle this, we define two peak states: ambiguous and unambiguous. A peak can be in only one state. An ambiguous or overlapped peak allows multiple transitions, while an unambiguous peak allows only one in- and one out-transition. Ambiguous peaks allow paths to share peaks subject to a penalty. The number of in- and out-transitions for these peaks are equal because peaks can only be created or destroyed in the ways allowed by our model. To limit the number of possible paths, only consecutive transitions are allowed for ambiguous peaks. A peak that corresponds to noise is modeled implicitly. Noise peaks are those not assigned to any path. The chemical shift mapping problem is defined as follows.

**Definition 2**. *The *mappings *for peak h_i _*∈ *T*_*k*-1 _*is the set of its possible residues R*(*h_i_*). *If *|*R*(*h_i_*)| > 1, *or if *|*R*(*h_i_*)| = 1 *and R*(*h_i_*) ∩ *R*(*h_l_*) ≠ ∅ *for h_l _*≠ *h_i_*, *then R*(*h_i_*) *is *ambiguous. *This set is obtained by first finding M*, *the maximum weighted k*-*dimensional matching on the graph defined by the above peak walking model that allows for subpaths and vertices to be shared*. *Let S be the amino acid sequence of the protein*, *and one-to-one function f*_0 _: *T*_0 _→ *S be the known reference assignment. For paths in M that end in some h_j _*∈ *T*_0 _*and h_i _*∈ *T*_*k*-1_, *add f*_0_(*h_j_*) *to R*(*h_i_*).

The optimal and near optimal sets of paths are generated to obtain different mappings per peak. This is done by modeling the problem as a binary integer linear program (BIP) and using the one-tree algorithm [29] to generate multiple solutions that are guaranteed to be within a given percentage of the optimal solution. This percentage, called the gap, is an input to the BIP solver. We used CPLEX^® ^as the solver.

### Mathematical model for peak walking

A linear objective function is maximized subject to linear constraints and binary variables.

#### Binary variables

The variables indicate the transitions and peak states.

• *X*_*hih*' _Equals to 1 if peak *h *∈ *T_i _*transitions to *h*' ∈ *T*_*i*+1_. This variable represents a consecutive transition.

• *X_hi _*Equals to 1 if *h *∈ *T_i _*is a single unambiguous peak. Equals to 0 if it is an ambiguous peak. This variable represents peak state.

• *D_hi _*Equals to 1 if *h *∈ *T_i _*is missing its peaks in *T_j_*, ∀*j *>*i*. This represents a peak that disappears and no longer reappears.

• *J_hih_' *Equals to 1 if *h *∈ *T_i _*is missing in *T*_*i*+1_, but transitions to *h*' ∈ *T*_*i*+2_. This represents a jump.

• *N_hi _*Equals to 1 if *h *∈ *T_i _*has no associated peaks in *T_j_*, ∀*_j _*<*i*. This represents a new peak.

#### Objective function coefficients

The objective function coefficients score the transitions and peak states, so the sum of the coefficients multiplied by their corresponding variables gives the score of the paths. Ideally, if a database of peak lists and chemical shift mappings are available, these coefficients could be obtained through training with machine learning techniques, so that the manual mapping process could be modeled. Unfortunately this database does not exist, so we used our best judgement to scale the scores relative to each other.

• $C\left(X_{hih^{\prime }}\right)=\Phi \left(\Delta \delta_{N}\left(h^{\prime },h\right),0,\text{tolN}\right)+\Phi \left(\Delta \delta_{H^{N}}\left(h^{\prime },h\right),0,\text{tolHN}\right)$. This is the score of a consecutive transition, where Φ(*x*, *m*, *s*) = 2 × (1 - *cdf*(*x*, *m*, *s*)). *cdf *is the cumulative distribution function of a normally distributed variable with mean *m *and standard deviation *s*. tolN and tolHN were set to values, such that *t_N _*and $t_{H^{N}}$, respectively, correspond to 2 standard deviations from a mean value of 0. The score is a number between 0 and 1, with small chemical shift changes being closer to 1 (because *x *is positive, so *cdf *returns a value of at least 0.5).

• $C\left(X_{hi}\right)=2\times \left(k-i-1\right)\times \left(\Phi \left(\frac{3t_{N}}{4},0,\text{tolN}\right)+\Phi \left(\frac{3t_{H^{N}}}{4},0,\text{tolHN}\right)\right)$. This score penalizes ambiguous peaks by rewarding unambiguous peaks. We require ambiguous peaks to have at least 2 paths of compensating transitions from *i *to *k *- 1. The reward decreases with increasing *i *because there are fewer transitions available. The $\frac{3}{4}$ inside Φ encourages the compensating transitions to have scores better than this.

• $C\left(D_{hi}\right)=\Phi \left(t_{N},0,\text{tolN}\right)+\Phi \left(t_{H^{N}},0,\text{tolHN}\right)$. This is the score for disappearing peaks. We give such peaks a positive score similar to a consecutive transition with a chemical shift change of *t_N _*and $t_{H^{N}}$.

• $C\left(J_{hih^{\prime }}\right)=0.75\times \left(\Phi \left(\Delta \delta_{N}\left(h^{\prime },h\right),0,\text{tolN}\right)+\Phi \left(\Delta \delta_{H^{N}}\left(h^{\prime },h\right),0,\text{tolHN}\right)\right)$. This is the score for jumps. The 0.75 encourages consecutive transitions over jumps of the same chemical shift change.

• $C\left(N_{hi}\right)=-\left(k-i-1\right)\times \left(\Phi \left(\frac{3t_{N}}{4},0,\text{tolN}\right)+\Phi \left(\frac{3t_{H^{N}}}{4},0,\text{tolHN}\right)\right)$. This is the score for new peaks. The score is negative to ensure that there must exist compensating transitions from *i *to *k *- 1.

• Peaks corresponding to noise have no transitions, and they get set to unambiguous because we are maximizing and the unambiguous score is non-negative.

#### Constraints

1. For each peak (ambiguous or unambiguous), the number of in-edges is equal to the number of out edges. Even if a peak disappears permanently (an out-edge), the peak must have come from a previous transition or be a new peak, which is considered an in-transition. From Figure 2, we can see that this constraint is ∀*i *∈ [1, *k *- 2], ∀*h *∈ *T_i_*, $\sum_{h^{\prime }}X_{h^{\prime }\left(i-1\right)h}+\sum_{h^{\prime }}J_{h^{\prime }\left(i-2\right)h}+N_{hi}=\sum_{h^{\prime }}X_{hih^{\prime }}+D_{hi}+\sum_{{}_{h^{\prime }}}J_{hih^{\prime }}$.

2. Ambiguous peaks are limited to only consecutive transitions. To get rid of jumps, define the *reified *constraint $J_{hi}=1↔\sum_{h^{\prime }}J_{h^{\prime }\left(i-2\right)h}\geq 1$, ∀*i *∈ [2, *k *- 1], ∀*h *∈ *T_i_*, where *J_hi _*is a binary variable. Then jumps are removed with *J_hi _*≤ *X_hi _*since if *X_hi _*= 0 (ambiguous), then *J_hi _*= 0 and $\sum_{h^{\prime }}J_{h^{\prime }\left(i-2\right)h}=0$. Disappearing and new peaks are handled similarly. Reified constraints allow one to get the truth value of a logical condition. Such conditions can be combined to form logical constraints, such as AND, OR, NOT, IF THEN, and even the absolute value of a linear expression. Reified constraints and logical constraints can be expressed as linear constraints using auxiliary binary variables and techniques from operations research [30].

3. For each unambiguous peak, the number of in-transitions is bounded above by 1; similarly for out-transitions. Define the reified constraints $I_{hi}=1↔\sum_{h^{\prime }}X_{h^{\prime }\left(i-1\right)h}+\sum_{h^{\prime }}J_{h^{\prime }\left(i-2\right)h}+N_{hi}\leq 1$, and $O_{hi}=1↔\sum_{h^{\prime }}X_{hih^{\prime }}+D_{hi}+\sum_{h^{\prime }}J_{hih^{\prime }}\leq 1$. Then the constraint is expressed as *I_hi _*= *X_hi _*and *O_hi _*= *X_hi_*. This, combined with Constraint 2, also handles, for ambiguous peaks, the constraint that the number of consecutive in-transitions is greater than 1 and the number of consecutive out-transitions is greater than 1.

4. Consecutive transitions generally do not zig-zag. That is, peaks typically do not take a large step in one direction and then take a large step in the reverse direction. To enforce this, let *h *∈ *T_i_*, *h*' ∈ *T*_*i*+1_, *h*″ ∈ *T*_*i*+2_. If 0.5 ≤ Δ*δ_N_*(*h*, *h*') ≤ *t_N_*, $0.05\leq \Delta \delta_{H^{N}}\left(h,h^{\prime }\right)\leq t_{H^{N}}$, 0.5 ≤ Δ*δ_N_*(*h*', *h*″) ≤ *t_N_*, $0.05\leq \Delta \delta_{H^{N}}\left(h^{\prime },h^{\prime \prime }\right)\leq t_{H^{N}}$, then consider the following vectors: $V_{hh^{\prime }}=\left(\delta_{N}\left(h^{\prime }\right)-\delta_{N}\left(h\right),10\left(\delta_{H^{N}}\left(h^{\prime }\right)-\delta_{H^{N}}\left(h\right)\right)\right)$ and $V_{h^{\prime }h^{\prime \prime }}=\left(\delta_{N}\left(h^{\prime \prime }\right)-\delta_{N}\left(h^{\prime }\right),10\left(\delta_{H^{N}}\left(h^{\prime \prime }\right)-\delta_{H^{N}}\left(h^{\prime }\right)\right)\right)$. The consecutive transitions *h *to *h*' to *h*″ zig-zag if the angle between *V*_*hh*' _and *V*_*h*'*h*″_, *θ*_*hh*'*h*″_, is between 105 and 180 degrees. When *h *transitions to *h*', transitions from *h*' to *h*″ that result in zig-zag are prevented by adding the constraint *X*_*hih*' _≤ *Z*_*h*'(*i*+1)_, where we have the reified constraint $Z_{h^{\prime }\left(i+1\right)}=1↔\left(\sum_{h^{\prime \prime }|\theta_{hh^{\prime }h^{\prime \prime }}\in \left[105,180\right]}X_{h^{\prime }\left(i+1\right)h^{\prime \prime }}=0\right)$. Thus, if *X*_*hih*' _= 1, then all consecutive transitions from *h*' to *h*″ that cause zig-zag are prevented because the sum is forced to 0.

#### Number of solutions

The number of solutions generated is dependent on the gap tolerance provided to CPLEX. Unless specified otherwise, a gap of 1% was used. To determine the number of solutions that should be generated, various numbers were tested to determine their effect on the average number of residues predicted per peak. We observed that as the number of solutions increased, the average number of residues plateaus, so we used the value at the start of the plateau as the number of solutions. Likely, no new mappings were generated because paths containing these mappings caused a violation of the gap optimality criteria.

#### Greedy peak walking

For comparison purposes, we implemented the greedy approach in NvMap, but also added no zig-zagging as described above, and jump handling of arbitrary length by allowing unmatched peaks in *T_i _*to be matched to peaks in *T_j _*for any *j *>*i*. The same chemical shift thresholds as those used by PeakWalker were used. None of the existing approaches deal directly with ambiguous mappings. To generate these without generating many mappings per peak, we used a greedy approach. For *h_i _*∈ *T*_*k*-1_, where *h_i _*is matched to *h_j _*∈ *T*_0_, in increasing order of Δ*δ_NH_*(*h_j_*, *h_b_*) for any *h_b _*≠ *h_i _*in *T*_*k*-1_, add *f*_0_(*h_j_*) to *R*(*h_b_*) until a maximum number of additional mappings have been added. Various values for the maximum were tested.

### Resonance assignment

Some definitions are needed before we can formally define this problem and present our algorithm.

**Definition 3**. *A NOESY peak p *(*δ_N_*(*p*), $\delta_{H^{N}}\left(p\right)$, *δ_H_*(*p*)) induces *an H^α ^peak for HSQC peak *$h\left(\delta_{N}\left(h\right),\delta_{H^{N}}\left(h\right)\right)$* if *Δ*δ_N_*(*p*, *h*) ≤ *σ_N_*, $\Delta \delta_{H^{N}}\left(p,h\right)\leq \sigma_{H^{N}}$, *and δ_H_*(*p*) *matches within 3 standard deviations of the mean value of *$\delta_{H^{\alpha }}\left(T\left(a\right)\right)$* of at least one amino acid a *∈ *R*(*h*), *where T*(*a*) *is the amino acid type of a*. *The mean and standard deviations of each amino acid type were obtained from the Biological Magnetic Resonance Data Bank (BMRB) *[31]. *σ_N_*, $\sigma_{H^{N}}$* are match tolerances*. *We used 0.5, 0.05 ppm. Since the intensity of NOESY peaks is inversely proportional to the distance of the underlying protons in contact, and intra-residue H^N^*, *H^α ^*'*s are relatively close, we can expect the intensity of intra-residue H^N^*-*H^α ^NOESY peaks to be large*. *Among the 8 closest (by *Δ*δ_NH_*(*p*, *h*)*) NOESY*-*induced H^α ^peaks of HSQC peak h*, *we took the 4 most intense peaks as a possible induced *$\delta_{H^{\alpha }}\left(h\right)$.

**Definition 4**. *A *contact *c consists of *$c\left[0\right]=H_{a}^{N}$ , *which is the amide proton of one amino acid denoted by a*, *and *$c\left[1\right]=H_{b}^{N}$*or *$H_{b}^{{}^{\alpha }}$ , *the amide or alpha proton of another amino acid denoted by b*. *For H^α^*, *it is possible that a *= *b*. *Let P*(*c*) *be the proton type (H^N^or H^α^) of c*[1].

**Definition 5**. *A *NOESY peak match *n consists of *$n\left[0\right]=\left(\delta_{N}\left(s\right),\delta_{H^{N}}\left(s\right)\right)$* of HSQC peak s*, $n\left[1\right]=\delta_{H^{N}}\left(t\right)$* or an induced *$\delta_{H^{\alpha }}\left(t\right)$* of HSQC peak t*, $n\left[2\right]=\left(\delta_{N}\left(p\right),\delta_{H^{N}}\left(p\right),\delta_{H}\left(p\right)\right)$* of some NOESY peak p; where*, Δ*δ_N_*(*s*, *p*) ≤ *σ_N_*, $\Delta \delta_{H^{N}}\left(s,p\right)\leq \sigma_{H^{N}}$, *and *Δ*δ_H_*(*t*, *p*) ≤ *σ_H_*. *We used 0.05 ppm for σ_H_*. *For H^α^*, *it is possible that s *= *t*. *Let P*(*n*) *be the proton type of n*[1].

**Definition 6**. *Amino acid *a matches *HSQC peak h if a *∈ *R*(*h*).

**Definition 7**. *Contact c *matches *NOESY peak match n if i) a *∈ *R*(*s*), *where amino acid a *∈ *c*[0] *and peak s *∈ *n*[0] , *ii) b *∈ *R*(*t*), *where b *∈ *c*[1]*and t *∈ *n*[1] , *and iii) P*(*c*) = *P*(*n*).

**Definition 8**. *Let C be the set of all contacts from the 3D structure of a homologous protein, let P be the set of all NOESY peaks, and let S be the amino acid sequence of the protein. The *resonance assignment *is a one-to-one function g*_1 _: *T*_*k*-1 _→ *S*, *where g*_1_(*h_i_*) ∈ *R*(*h_i_*) *for all h_i _*∈ *T*_*k*-1_, *and the NOE assignment is a one-to-one function g*_2 _: *P *→ *C*, *such that the scoring function *$\left(\sum_{h_{i}\in T_{k-1}}\sum_{g_{1}\left(h_{i}\right)\in S}w_{1}\left(h_{i},g_{1}\left(h_{i}\right)\right)\right)+\left(\sum_{p\in P}\sum_{g_{2}\left(p\right)\in C}w_{2}\left(p,\;g_{2}\left(p\right)\right)\right)$* is maximized*. *The functions *$w_{1}:T_{k-1}\times S\to \mathbb{R}$*and w*_2 _: *P *× *C *→ ℝ *weigh each individual resonance and NOE assignment, respectively*.

The BIP from our previous work [19] was modified to support the NOE assignment of H*^N^*-H*^N ^*and H*^N^*-H*^α ^*contacts without TOCSY.

#### Binary variables

The variables indicate individual resonance and NOE assignments. Note that each NOESY peak will be assigned to at most one NOESY peak match and vice versa. Therefore, assigning contacts to NOESY peak matches is equivalent to assigning contacts to NOESY peaks. If there is only one possible NOESY peak match for a given NOESY peak, then that peak is unambiguous.

• *X*_*a*,*h *_Equals to 1 if amino acid *a *is assigned to HSQC peak *h*, where *a *matches *h*.

• *X*_*c*,*n *_Equals to 1 if contact *c *is assigned to NOESY peak match *n*, where *c *matches *n*.

#### Objective function coefficients

A linear objective function is maximized. The coefficients are the weights of the assignments, and they are non-negative.

• $w_{1}\left(X_{a,h}\right)=3\times \left(1-\frac{\Delta \delta_{NH}\left(f_{0}^{-1}\left(a\right),h\right)-min\left(h\right)}{max\left(h\right)-min\left(h\right)}\right)$. This is the score of assigning amino acid *a *with reference peak $f_{0}^{-1}\left(a\right)$ to target peak *h*. *min*(*h*) and *max*(*h*) is the smallest and largest, respectively, Δ*δ_NH _*among the amino acids in *R*(*h*).

• $w_{2}\left(X_{c,n}\right)=\Phi \left(\Delta \delta_{N}\left(p,s\right),0,\frac{\sigma_{N}}{2}\right)+\Phi \left(\Delta \delta_{H^{N}}\left(p,s\right),0,\frac{\sigma_{H^{N}}}{2}\right)+\Phi \left(\Delta \delta_{H}\left(p,t\right),0,\frac{\sigma_{H}}{2}\right)+F\left(c\right)$. This is the score of assigning contact *c *to NOESY peak match *n*, where HSQC peak *s *∈ *n*[0], HSQC peak *t *∈ *n*[1], and NOESY peak *p *∈ *n*[2]. *F*(*c*) is a weight on the type of contact. In the absence of missing NOESY peaks, contacts involving adjacent amino acids should have a NOESY peak match, so it is natural for adjacent amino acid contacts to have higher weight than nonadjacent. Φ is the same as the one defined in the peak walking mathematical model.

#### Constraints

1. Each amino acid *a *is assigned to at most one HSQC peak. This is $\sum_{h}X_{a,h}\leq 1$.

2. Each HSQC peak *h *is assigned to at most one amino acid. This is $\sum_{a}X_{a,h}\leq 1$.

3. Each contact *c *is assigned to at most one NOESY peak match. This is $\sum_{n}X_{c,n}\leq 1$.

4. Each NOESY peak *p *∈ *n*[2] of NOESY peak match *n *is assigned to at most one contact. This is $\sum_{c,n\left[0\right],n\left[1\right]}X_{c,n}\leq 1$.

5. Each pair of HSQC peaks *n*[0], *n*[1] of NOESY peak match *n *has at most one NOESY peak. This is $\sum_{c,n\left[2\right]}X_{c,n}\leq 1$.

6. Contact *c *is assigned to NOESY peak match *n *if and only if amino acid *c*[0] is assigned to HSQC peak *n*[0], and *c*[1] is assigned to *n*[1]. This constraint is similar to the if and only if constraint in our previous work.

(a) $\forall c,\forall h,\sum_{n|h\in n\left[0\right]}X_{c,n}\leq X_{c\left[0\right],h}$

(b) $\forall c,\forall h,\sum_{n|h\in n\left[1\right]}X_{c,n}\leq X_{c\left[1\right],h}$

7. Each H*^α ^*proton, *z_a _*of amino acid *a*, is assigned to at most one induced H*^α ^*peak, *y_h _*of HSQC peak *h*. Let $b_{z_{a,yh}}=1↔\sum_{c,n\;|\;z_{a}\in c\left[1\right],y_{h}\in n\left[1\right]}X_{c,n}\geq 1$ be a reified constraint, where $b_{z_{a,yh}}=1$ if *z_a _*is assigned to *y_h_*. The summation is over all *X_c,n _*that contain *z_a _*and *y_h_*. The constraint is then $\forall z_{a},\sum_{y_{h}}b_{z_{a},yh}\leq 1$.

8. Each induced H*^α ^*peak, *y_h _*of HSQC peak *h*, is assigned to at most one H*^α ^*proton. This constraint is $\forall y_{h},\sum_{z_{a}}b_{z_{a},yh}\leq 1$.

#### Multiple assignment possibilities

Similar to PeakWalker, multiple solutions corresponding to different assignment possibilities were generated. From the multiple solutions, a consensus assignment was generated by running the above BIP with *w*_1_(*X_a,h_*) equal to the number of times amino acid *a *was assigned to peak *h *and *w*_2_(*X_c,n_*) equal to the number of times contact *c *was assigned to NOESY peak match *n*.

### PeakWalker results

Table 1 compares the accuracy between the greedy algorithm with PeakWalker. Different values for the maximum number of candidate residues were tested with greedy. Only a select few are shown. Accuracy is defined as the number of target peaks whose possible mappings contain the correct residue divided by the number of peaks with mappings predicted, including noise peaks. Since one could predict mappings for only a few peaks and still have high accuracy, we have also included the number of peaks whose mappings contain the correct residue. The numbers are averages over 10 trials, where each trial used different noise peaks. The average number of residues predicted per peak varied by at most 0.1 in the trials (not shown). For Histone H1, the accuracy for the ambiguous peak list case is defined as the number of target peaks whose mappings include all the possible residues divided by the number of peaks with mappings. In general, PeakWalker has comparable or better accuracy, and comparable or more correct predictions with fewer candidate residues per peak.

**Table 1:** Comparison between Greedy and PeakWalker

Protein	Method	Num Correct	Num Correct Range	Acc (%)	Acc Range (%)	Avg Num Res/Peak
hBcl_XL_	Greedy	110.9	110-111	95.7	94-96.5	1
	Greedy	111.1	111-112	90.7	89.5-91.7	1.7
	PeakW	116.3	116-117	96.8	95.9-97.5	1.4

Ubch5b	Greedy	114.6	113-115	94.2	91.5-96.7	1
	Greedy	116.9	116-118	94.4	93.5-95.2	1.2
	Greedy	120.8	120-122	97.2	96-98.4	1.5
	PeakW	120.4	119-123	98.1	96.0-99.2	1.2

Histone H1*^U^*	Greedy	78.1	76-83	91.4	89.4-93.3	1
	Greedy	83.0	83-83	95.5	94.3-96.5	1.5
	PeakW	85.1	85-86	99.3	97.7-100	1.3

Histone H1*^A^*	Greedy	72.0	72-72	82.8	80.9-83.7	2.0
	PeakW	76.0	76-76	88.8	87.4-89.4	1.3

The numbers are averages over 10 trials. Accuracy is defined as the number of target peaks whose mappings contain the correct residue divided by the number of peaks with mappings, including noise peaks. Avg Num Res is the average number of residues predicted per peak with a mapping. Results for the best guess unambiguous mapping (*U*) and the ambiguous mapping (*A*) are given for Histone H1. The mappings for a peak is correct in the ambiguous case if it contains all possible residues.

The peak lists of hBcl_XL _contained the most errors among the proteins. Out of 136 peaks in the reference, only 114 had a complete path without any missing peaks between the reference and target. 12 residues did not have any peak in the reference list, but had peaks in the other lists. There was one residue with a jump of length 2, and 3 residues with a jump of length 3. There were no jumps longer than 3. Despite not explicitly modeling jumps of length 3, on average PeakWalker got 2.4 of those mappings correct. For UbcH5B, all the target peaks had corresponding peaks in the reference. There were 2 jumps of length 2, and 4 jumps of length 3. On average, PeakWalker got 3.2 out of those 4 correct. There were no jumps in histone H1.

We also tested hBcl_XL _using only 6 peak lists instead of 11 by taking every other list. This corresponds to performing fewer NMR experiments. The accuracy decreased slightly to 95.7% with 114.9 correct predictions. hBcl_XL _was also tested with no overlapped peaks merged in the input. This corresponds to the result if all overlapped peaks could be predicted. For this test, at a cost of optimality, the gap was set to 4% to keep the run time to less than 5 mins per trial on an Intel Core 2 Duo T9300 laptop with 3 GB RAM. Nevertheless, the accuracy was 98.7% with an average number of correct mappings of 138.0 (an increase of over 21), at an average of 1.7 residues per peak. This indicates that peak overlap can hide many peak mappings, which can be a problem if these residues are involved in binding. However, binding residues tend to have chemical shift changes upon binding, so to completely hide such a residue, every time it moves there must exist at least another peak with similar chemical shift to overlap it. In the case of hBcl_XL_, peak overlap masked only the target peak of one known binding residue with significant shift changes, but the residue's other peaks were not masked.

Table 2 displays the results of a noise test on hBcl_XL_. The results are averages over 10 trials. The number of noise peaks added ranged from 0 to 50% of the number of peaks prior to addition. All the tests in Table 1 had 10% noise. The accuracy at 10% is actually slightly larger than the accuracy at 0% because by chance, some noise peaks provided alternative paths from the target peak to its correct reference. Accuracy depends on the location of the noise peaks relative to non-noise peaks. In general, the number of correct predictions and the accuracy decreases with increasing noise, but the decrease is relatively graceful for randomly distributed noise.

**Table 2:** Results for PeakWalker on hBcl_XL _with various noise levels

Noise (%)	Num Correct	Num Correct Range	Acc (%)	Acc Range (%)
0	116	116-116	96.7	96.7-96.7
10	116.3	116-117	96.8	95.9-97.5
20	115.8	115-117	95.8	95-97.5
30	115.5	114-116	94.9	91.9-97.5
40	115.2	114-116	95.3	93.4-96.6
50	115.2	113-117	93.5	91.1-95.1

The results are averages over 10 trials.

### PeakAssigner results

To compare the combined NOE and resonance assignment approach of PeakAssigner with the method in our previous work, we ran both on data simulated from the structures [PDB:1KA5], [PDB:1EGO], [PDB:1G6J], [PDB:1SGO], and [PDB:1YYC], which were part of the test set in our previous work. Rather than using the simulated data provided by the authors of the CR method, which was done in our previous work, we simulated the data ourselves so that we could trace the results back to the data. In this test, the mappings of all peaks contained the correct residue and the H*^α ^*assignments were known. NOESY peaks were simulated using chemical shift data from the protein's BMRB entry: [BMRB:2030] for 1KA5, [BMRB:491] for 1EGO, [BMRB:5387] for 1G6J, [BMRB:6052] for 1SGO, and [BMRB:6515] for 1YYC.

The results are given in Table 3. Each PDB file contained multiple 3D models. The table shows the average result from using every pair of structures, where one was the template structure and the other was the target. The noise level is defined as the number of NOESY peak matches divided by the number of contacts. With the exception of 1G6J, which has a low noise level, our new method was better, especially when the noise level increased. We also tested 1SGO with different noise levels by using different values for the match tolerance (data not shown). For a noise level of 4.6, the old method was 0.5% more accurate, but for noise levels from 5.5 to 10.3, the new method did 0.2 to 4.2% better. Larger proteins typically have higher noise levels due to increased peak overlap.

**Table 3:** Comparison between the old assignment method in [19] and the new method

PDB ID	1KA5	1EGO	1G6J	1SGO	1YYC
Noise (X)	5.6	5.3	3.8	8.2	7.6
Acc New (%)	100	95.6	93.5	87.9	95.2
Range New (%)	100	89.9-100	93.1-94.4	81.8-99.3	90-99.4
Acc Old (%)	100	92.8	94.4	86.7	89.4
Range Old (%)	100	89.9-97.5	94.4-94.4	76.7-96.3	75.5-96.8
NOE Acc (%)	92.6	89.0	94.2	86.6	89.8
Range NOE (%)	91.0-94.0	79.4-94.7	92.3-96.2	81.8-93.5	84.9-94.2

The noise level is defined as the number of NOESY peak matches divided by the number of contacts. The accuracy is the number of correct one-to-one mappings divided by the number of mappings. NOE assignment accuracy is the number of correct NOESY peak to contact assignments divided by the number of assignments. The NOE assignment accuracy is only for the new method because the old method does not do NOE assignment.

Table 4 shows the assignment results for hBcl_XL_, UbcH5B, and histone H1. The values are averages over 10 trials, where each trial is a different NOESY peak simulation. Peak mappings were obtained from PeakWalker, and the unambiguous reference mapping was used to measure the accuracy on histone H1. As expected, the resonance assignment accuracies were slightly less than those for the input many-to-one mappings. However, the number of correct assignments for hBcl_XL _and histone H1 was less than expected when comparing to Table 1. This is likely due to differences between the contacts in the template and target structures. Their superpositions were greater than that for UbcH5B, and the templates had fewer residues than the target. When we used the target as the template structure for resonance assignment, the number of correct assignments increased to 110.8 for hBcl_XL _and 72.3 for histone H1. Other types of errors, such as missing NOESY peaks, had only a small influence on the number of correct assignments. Another factor is that our accuracy definition did not take into account peaks that were assigned to the wrong amino acid, but have almost identical chemical shift to the correct target peak of that amino acid. When this is taken into account, the number of correct assignments increased by about 2.6 for hBcl_XL_. There was no change for histone H1 because its peak lists had no overlapped peaks.

**Table 4:** One-to-one resonance assignment results from PeakWalker input

Protein	UbcH5B	Histone H1	hBcl_XL_	hBcl_XL_*
Num Correct	119.5	66.2	101.9	99.8
Num Correct Range	119-120	65-67	101-103	99-101
Acc (%)	98.0	94.7	95.6	94.5
Acc Range (%)	97.5-98.4	92.9-95.7	94.4-97.2	93.5-95.2
Num H*^N ^*- H*^N ^*Correct	157	114	116.1	118.3
Acc H*^N ^*- H*^N ^*(%)	92.7	90.9	90.0	86.3
Num H*^N ^*- H*^α ^*Correct	168.2	104.9	128.6	0
Acc H*^N ^*- H*^α ^*(%)	75.6	65.4	63.1	0

The input many-to-one mappings for hBcl_XL _had a 96.7% accuracy with 116 correct and 1.3 residues per peak on average. The input for UbcH5B had values of 98.3%, 120, and 1.2. The input for histone H1 had values of 98.8%, 85, and 1.3. The results are averages over 10 trials, where each trial is a different NOESY simulation. The unambiguous reference mapping was used to measure the accuracy for histone H1. The NOE assignment accuracy for each contact type is defined as the percentage of the number of contacts of the given type that is assigned to the correct NOESY peak. * The last column gives the results of using only H*^N^*-H*^N ^*contacts for hBcl_XL_.

Despite using ambiguous induced H^*α *^chemical shift assignments for each HSQC peak, the accuracies of the H*^N^*-H*^α ^*assignments are over 60%, even with a 5% H*^α ^*missing rate. Nevertheless, the results for hBcl_XL _that used only H*^N^*-H*^N ^*indicate that resonance assignment accuracy is not necessarily impacted significantly if H*^α ^*is not used.

Table 5 shows the resonance assignment results for hBcl_XL _with different many-to-one input mappings. When the number of candidate residues per peak increased, the accuracy and the number of correct assignments decreased. However, the decrease was much more pronounced for the input with poorer accuracy. The decrease in the other case was minimal. Thus, erring on producing extra possible mappings is less detrimental if it can be done accurately.

**Table 5:** One-to-one resonance assignment results for hBcl_XL _with different input many-to-one mappings

Num Correct Input	111/123	111/123	111/115	111/115
Avg Num Res/Peak	2.3	3.3	2	3
Num Correct Output	92.2	86.3	95.1	94.3
Num Correct Range	90-95	80-93	93-97	93-96
Acc (%)	91.8	86.5	94.6	94.3
Acc Range (%)	89.1-95.0	79.2-93.0	92.1-96.9	92.1-96.0

The results are averages over 10 trials, where each trial is a different NOESY simulation.

**Figure 3:**
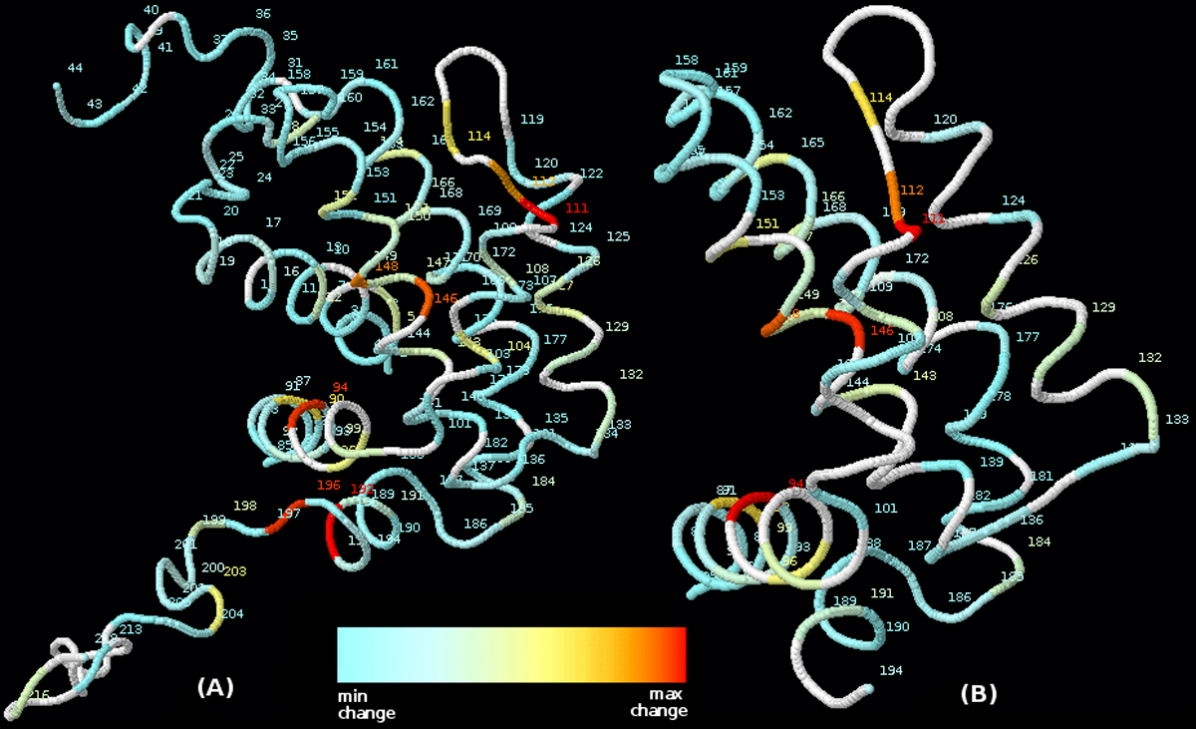
**The chemical shift changes for the residues in hBcl_XL_. **(A) gives the known shift changes and the structure from which the NMR data was derived. (B) gives the shift changes from resonance assignment and the input structure for assignment. Residues are labeled by their residue number. Unassigned residues are unlabeled and colored white.

**Figure 4:**
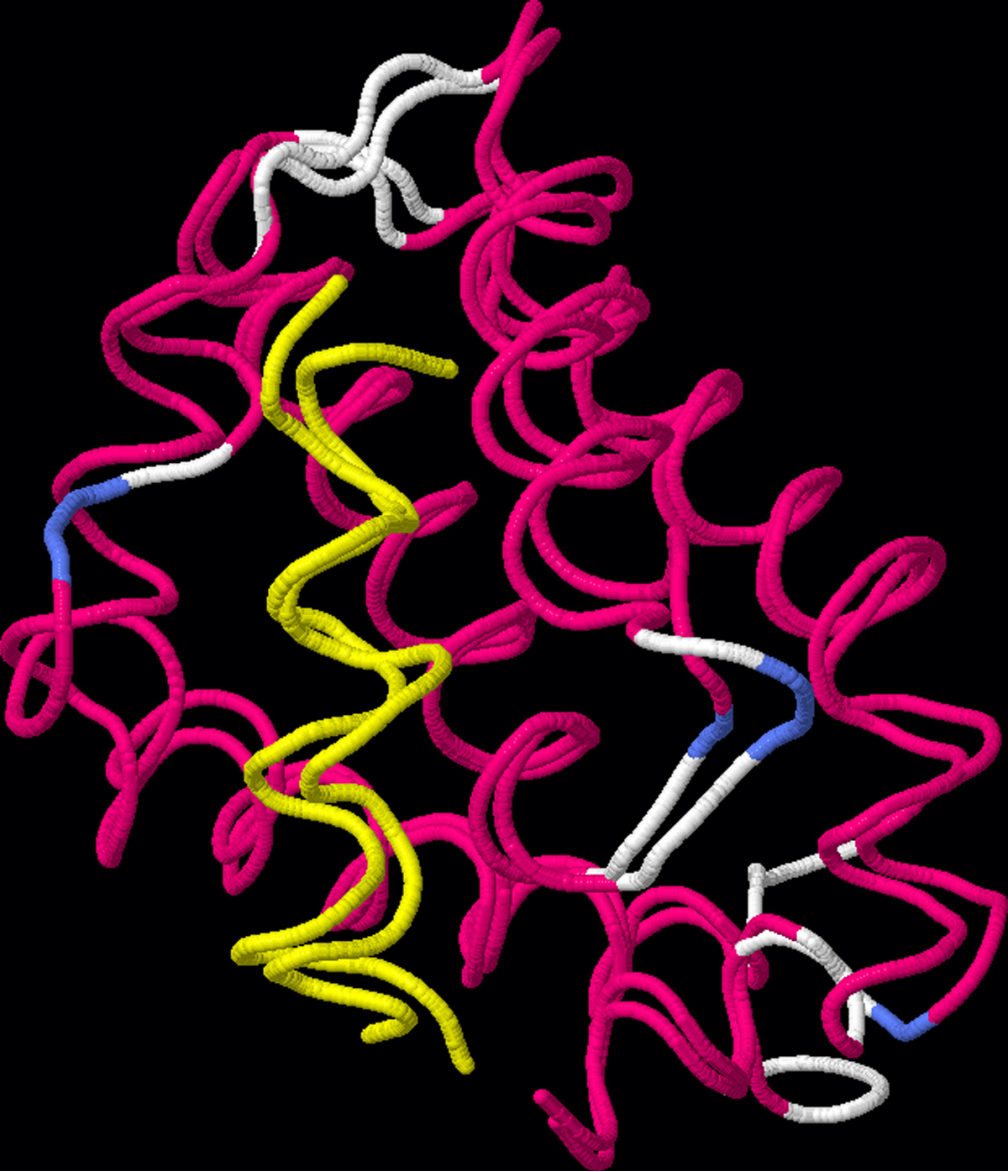
**Structure alignment of hBcl_XL_-Bak protein-protein complex with [PDB:**1BXL**].** The complex was obtained by docking the Bak peptide (yellow) in [PDB:1BXL] to the homology model for hBcl_XL _using putative binding residues 90, 94, 111, 112, 114, 146, 148, and 192 as constraints. ClusPro [39,40] was used for protein-protein docking, where the lowest energy structure from the largest cluster was used. MM-align [41] was used for structure alignment.

Once resonance assignment is performed, one can compute the chemical shift change between each target peak and its assigned reference peak. Residues with large changes might indicate their involvement in binding. Figure 3 shows the chemical shift changes of the residues of hBcl_XL_. For this protein, residues with large changes are involved in binding or near binding residues, but this is not always the case for all proteins because changes can also be attributed to allosteric changes. Except for 2 residues involved in binding, the reference solution and PeakAssigner agree. Residue 196 was not in the input structure for assignment, and the peak for 192 was not in the target peak list. However, 192 was correctly predicted as missing its peak by PeakWalker, and correctly predicted as having a large shift change using its peaks in the other peak lists. Figure 4 shows the result of docking the Bak peptide from 1BXL to the homology model for hBcl_XL _using the putative binding residues 90, 94, 111, 112, 114, 146, 148, and 192 as constraints. The binding affinity can be determined by computing the dissociation constant, which can be obtained from model fitting using the peak paths and the predicted paths according to some model of binding [11].

Even with NOE information, a one-to-one mapping for all residues is not always possible. Our approach, however, facilitates an iterative semi-automated approach. Once assignments and paths have been verified, perhaps using additional information, the variables corresponding to those peaks and residues can be removed from the BIPs, and then PeakWalker and PeakAssigner can be rerun. Both programs can return multiple near-optimal solutions to account for ambiguity.

## Conclusions

We also tested our method on the protein calmodulin to test the slow exchange case, and to identify problems for future work. Currently, we are not aware of any automated methods for slow exchange. In general, peak tracking is more difficult here because there are no intermediate peaks to track peak movements in increments, and the number of peaks in the spectra can be almost double the number in fast exchange.

We generated peak lists using chemical shifts in [BMRB:6541] (free form) and [BMRB:15624] (bound form). Four peak lists with saturation levels 0:1, 1:4, 3:4, and 1:1 were generated. Residues with backbone N and H*^N ^*chemical shifts in 6541 and 15624 within 0.5 ppm and 0.05 ppm were assumed to have only one peak rather than two. Peak intensities were generated based on the saturation levels. For the case with no noise peaks and no errors, except for 3 residues present in 6541, but not in 15624, the Greedy method performed poorly at less than 100 residues correct out of 143 with 7.6 peaks/residue. PeakWalker performed even worst, which is expected since both methods do not model slow exchange. Cutoffs of 2.0 ppm for the N chemical shifts and 0.4 ppm for H*^N ^*were used.

In the Methods section, we describe a mathematical model for slow exchange, which uses the peak intensities. Using an intensity cutoff of a 15% difference from the expected intensity ratio, the method got 132 correct at 5.7 peaks/residue. The 11 incorrect had chemical shift changes outside the 2.0, 0.4 ppm cutoffs. Unfortunately, calmodulin undergoes a large conformational change upon binding its target peptide (hinge motion in a long helix), and those 11 residues are important for binding and conformational change. A 4.0, 0.8 ppm cutoff would be needed to cover the chemical shift changes of all residues, but this will result in a prohibitive number of possible peaks per residue. Preliminary results of using an iterative approach of using both PeakWalker and PeakAssigner was successful only for the case with no errors, no noise, and no missing NOESY peaks (137 correct one-to-one mappings). Here, we used contact information from the free form structure [PDB:1EXR], we fixed residue-peak assignments supported by NOESY peaks and paths that occurred frequently, and we increased the cutoffs in increments. For the case with errors, which is the norm, additional NMR data, such as NOESY data for the contacts between the protein and ligand, will likely be needed to reduce ambiguity.

It would be ideal to automate 3D structure determination of the bound protein for proteins that can undergo conformational change upon binding under either fast or slow exchange using limited NMR data, and a 3D structure of the free form or a homologous structure of the bound form. Currently this is a very challenging computational problem that involves protein folding and flexible protein-ligand docking, while satisfying constraints derived from limited experimental data.

Drug screening is expensive in terms of both time and money. Although much progress remains to be made, our mathematical modeling approaches for automating chemical shift mapping using limited data are steps towards high-throughput NMR studies.

## Availability

The Java source code is available by request to the corresponding author.

## Methods

PeakWalker and PeakAssigner were tested on hBcl_XL_, UbcH5B, and histone H1. This section describes the test data and the errors that were introduced. The mathematical model for the slow exchange case is also given.

### Peak lists

The hBcl_XL _data set consisted of 11 peak lists. The reference peak list contained 148 peaks, while the target contained 142. UbcH5B consisted of 5 peak lists. The reference contained 127 peaks, while the target also contained 127. Histone H1 consisted of 2 peak lists. The reference contained 97 peaks, while the target contained 86. Unlike the other proteins, the assignment for Histone H1 was unknown, so we performed the chemical shift mapping manually to obtain a reference solution. Due to ambiguities inherent with chemical shift mapping, especially using only 2 peak lists, we produced both an ambiguous mapping, and for testing purposes, our best guess unambiguous mapping.

The peak lists of hBcl_XL_, UbcH5B, and histone H1 were edited to introduce errors. To obtain errors due to overlapped peaks, peaks within the same peak list that have Δ*δ_N _*≤ 0.1 ppm and $\Delta \delta_{H^{N}}\leq 0.01$ ppm were merged into a single peak. Such peaks would likely appear as a single peak when viewing the spectra. Multiple peaks could be merged into a single peak. Such merges in the target list will result in at most only one of the peaks being mapped. In hBcl_XL_, 5 residues had identical chemical shifts in the target list. After merging, hBcl_XL _had 136 peaks in the reference list and 122 in the target. UbcH5B had 127 in the reference and 123 in the target. There were no changes to the Histone H1 lists. To simulate noise peaks, in each peak list, we introduced noise peaks in the range of the N and H*^N ^*chemical shifts, 99-133 ppm and 6.25-10.75 ppm, respectively. Unless stated otherwise, the number of noise peaks added to each peak list is equal to 10% of its size prior to the addition.

### NOESY peak simulation

NOESY peaks were simulated using the contacts in the 3D structure (within 4.5Å), N and H*^N ^*chemical shift values from the target peak list, and H*^α ^*chemical shift values from either ShiftX predictions [32] or from the BMRB. For hBcl_XL_, we used the protein threading server LOMETS [33], to obtain a 3D structure. The structure chosen among the possibilities returned by LOMETS was the one that used [PDB:1LXL] as the threading template. It consisted of 178 residues after the flexible loop region was removed. ShiftX was used to obtain the H*^α ^*chemical shift values. For Ubch5b, we used the structure named "ubch5b-not4_1.pdb" that was provided with the peak lists, and ShiftX for the H*^α ^*chemical shifts. The structure consisted of 147 residues. For histone H1, we used [PDB:1UST] for the structure and [BMRB:6161] for the H^*α *^chemical shifts. It consisted of 92 residues.

A global offset to calibrate the N, H*^N ^*chemical shifts of the NOESY against the same shifts in the target HSQC is assumed to have already been obtained from a calibration step, so we simulated only local calibration errors. Local calibration noise, randomly distributed between 0 and 0.15 ppm for N, 0 and 0.015 ppm for H*^N^*, were introduced to NOESY peaks. Compared to resonance assignment, global calibration can be performed manually relatively quickly. Similar to our previous work, missing inter-residue contacts were introduced with the following probabilities (0, 0.05, 0.21, 0.41, 0.51) for contacts within the following distances (1.0, 2.0, 3.0, 4.0, 4.5)Å, respectively. Missing intra-residue H*^N^*-H^*α *^contacts were introduced with probability 0.05. With size 10% of the number of NOESY peaks, NOESY peaks corresponding to noise were added in the range 99-133 ppm for *N*, 6.25-10.75 ppm for *H^N^*, and 2-6 ppm for *H^α^*.

### Protein 3D structures

The 3D structure for NOESY peak simulation shall be referred to as the target structure. This structure corresponds to the NMR data and is unknown to the assignment algorithm. The homologous structure used as input to the assignment algorithm shall be referred to as the template structure. The homology-modeling server SWISS-MODEL [34-36] was used to obtain the templates. Reduce [37] was used to add the coordinates of hydrogen atoms to the templates. As input to SWISS-MODEL, the template used for hBcl_XL _was [PDB:3FDL]. It consisted of 154 residues. Residues 27 to 82 were not present in the file. The 3D superposition between the target and template is 13.6Å. However, if only residues 85-194 are considered, the structure alignment is 2.3Å according to the program CE [38]. The template for Ubch5b was [PDB:2ESK], which consisted of 147 residues. The superposition is 2.4Å, where all residues are aligned. The template for histone H1 was [PDB:1YQA], which consisted of 85 residues. The superposition is 4.9Å, but the structure alignment is 2.0Å, using residues 9-82.

### Mathematical model for slow exchange peak tracking

Similar to the fast exchange case, we model slow exchange as a k-dimensional matching problem. The difference is that we allow vertices in the graph to represent two peaks in addition to one; and in the scoring function, we consider for a pair of peaks their intensities relative to the concentration ratio of the protein and ligand.

**Figure 5:**
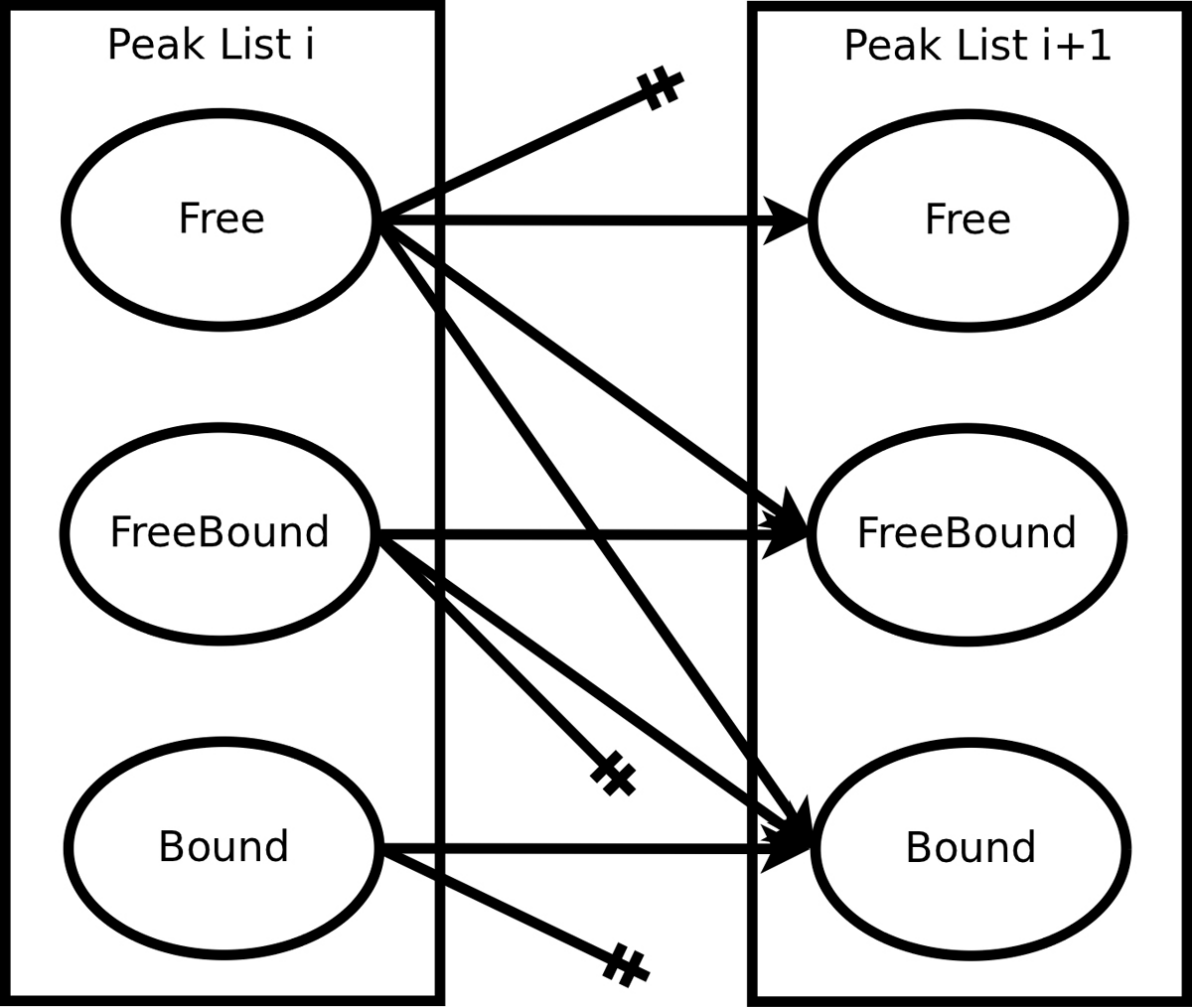
**Peak tracking model for slow exchange.** The free state corresponds to a residue in the free form. The freebound state corresponds to a residue exchanging between the free and bound forms, and the bound state corresponds to a residue in the bound form only. The arrows describe the possible transitions from each state. A transition with no arrow at the end corresponds to a residue missing its peaks in all subsequent peak lists.

We define 3 types of vertices based on 3 different peak/residue states. A *free *vertex represents a peak corresponding to a residue potentially in the free form. A *freebound *vertex represents a pair of peaks corresponding to the same residue in both the free and bound forms. A *bound *vertex represents a peak corresponding to a residue in the bound form only. Figure 5 illustrates the possible transitions from each state. From the free state, a residue can transition to any of the 3 states. From the freebound state, a residue can remain in this state or transition to the bound state. A residue in the freebound state cannot transition back to the free state. Once in the bound state, a residue must remain there. Initially, all peaks in the first peak list are in the free state. In the final peak list, we assume the protein is fully saturated with the ligand, so no residues are in the freebound state. We also allow a residue to transition to a missing state, where its peaks disappear in all subsequent peak lists. A missing transition from the freebound state means that both peaks are missing.

Similar to the fast exchange case, a linear objective function is maximized subject to linear constraints and binary variables.

#### Binary variables

The variables represent the transitions/edges between vertices, where each vertex represents a peak or a pair of peaks in some state and from some peak list.

• *X_hish's' _*Equals to 1 if peak *h *∈ *T_i _*in state *s *, where *s *∈{free, freebound, bound} or a pair of peaks *h_a_*, *h_b _*for *s *= split, transitions to peak *h*' ∈ *T*_i+1 _in state *s*' or a pair of peaks $h_{a}^{\prime },h_{b}^{\prime }$ for *s*' = split, where *s*' ∈ {free, freebound, bound, missing}. For *s*' = missing, *h*' is empty.

#### Objective function coefficients

The scores of the transitions depends on the states.

• $C\left(X_{hi\left[\text{free}\right]h^{\prime }\left[\text{free}\right]}\right)=\Phi \left(\Delta \delta_{N}\left(h^{\prime },h\right),0,0.25\right)+\Phi \left(\Delta \delta_{H^{N}}\left(h^{\prime },h\right),0,0.025\right)$, where Φ is the same as the one defined in the mathematical model for fast exchange.

• $C\left(X_{hi\left[\text{free}\right]{h^{\prime }}_{a}{h^{\prime }}_{b}\left[\text{freebound}\right]}\right)=\Phi \left(\Delta \delta_{N}\left(h_{a}^{\prime },h\right),0,0.25\right)+\Phi \left(\Delta \delta_{H^{N}}\left(h_{a}^{\prime },h\right),\;0,0.025\right)+\Phi \left(|\frac{I\left({h^{\prime }}_{a}\right)}{I\left({h^{\prime }}_{a}\right)+I\left({h^{\prime }}_{b}\right)}-R_{i}|,0,0.15\right)$, where *I*(·) gives the intensity of the given peak, *R_i _*is the expected intensity ratio based on the concentration ratio of ligand to protein, and $h_{a}^{\prime }$ is closer to *h *than $h_{b}^{\prime }$ is to *h *based on Δ*δ_NH_*.

• *C*(*X*_*hi*[free]*h*'[freebound]_) = 0.001. Since the chemical shift of *h*' can be very different from *h *for a given residue, we set this score to be a small constant.

• $C\left(X_{h_{a}h_{b}\left[\text{freebound}\right]{h^{\prime }}_{a}{h^{\prime }}_{b}\left[\text{freebound}\right]}\right)=\Phi \left(\Delta \delta_{N}\left(h_{a}^{\prime },h_{a}\right),0,0.25\right)+\Phi \left(\Delta \delta_{H^{N}}\left(h_{a}^{\prime },h_{a}\right),0,0.025\right)+\Phi \left(\Delta \delta_{N}\left(h_{a}^{\prime },h_{b}\right),0,0.25\right)+\Phi \left(\Delta \delta_{H^{N}}\left(h_{b}^{\prime },h_{b}\right),0,0.025\right)+\Phi \left(|\frac{I\left({h^{\prime }}_{a}\right)}{I\left({h^{\prime }}_{a}\right)+I\left({h^{\prime }}_{b}\right)}-R_{i}|,0,0.15\right)$, where $h_{a}^{\prime }$ is closer to *h_a _*than to *h_b_*.

• $C\left(X_{h_{a}h_{b}\left[\text{freebound}\right]{h^{\prime }}_{b}\left[\text{bound}\right]}\right)=\Phi \left(\Delta \delta_{N}\left(h_{b}^{\prime },h_{b}\right),0,0.25\right)+\Phi \left(\Delta \delta_{H^{N}}\left(h_{b}^{\prime },h_{b}\right),0,0.025\right)$, where $h_{b}^{\prime }$ is closer to *h_b _*than to *h_a_*.

• $C\left(X_{h_{b}\left[\text{bound}\right]{h^{\prime }}_{b}\left[\text{bound}\right]}\right)=\Phi \left(\Delta \delta_{N}\left(h_{b}^{\prime },h_{b}\right),0,0.25\right)+\Phi \left(\Delta \delta_{H^{N}}\left(h_{b}^{\prime },h_{b}\right),0,0.025\right)$

#### Constraints

• Define the following auxiliary variables for each vertex. $O_{his}=\sum_{h^{\prime }s^{\prime }}X_{hish^{\prime }s^{\prime }}$, which represents the sum of the variables corresponding to the out-edges from vertices that contain peak *h *∈ *T_i _*in state *s*. $I_{his}=\sum_{h^{\prime }s^{\prime }}X_{h^{\prime }\left[i-1\right]s^{\prime }hs}$, which represents the sum of the variables corresponding to the in-edges into vertices that contain peak *h *∈ *T_i _*in state *s*.

• The number of in-edges, and the number of out-edges is bounded by one to prevent path overlap. This is *I_his _*≤ 1 and *O_his _*≤ 1, respectively.

• Analogous to the fast-exchange case, we have the number of in-edges equal to the number of out-edges. This is *O_his _*= *I_his_*.

• Define the following auxiliary variables for each peak. $O_{hi}=\sum_{sh^{\prime }s^{\prime }}X_{hish^{\prime }s^{\prime }}$, which represents the sum of the variables corresponding to the out-edges from vertices that contain peak *h *∈ *T_i _*in any state. $I_{hi}=\sum_{h^{\prime }s^{\prime }s}X_{h^{\prime }\left[i-1\right]s^{\prime }hs}$, which represents the sum of the variables corresponding to the in-edges into vertices that contain peak *h *∈ *T_i _*in any state.

• Since a vertex can contain more than one peak, to ensure that each peak gets assigned to at most one state and path, we have *I_hi _*≤ 1, *O_hi _*≤ 1, and *O_hi _*= *I_hi_*.

## Competing interests

The authors declare that they have no competing interests.

## Authors' contributions

RJ, XG, and ML developed the ILP approaches. RJ wrote the code, performed the experiments, and wrote the manuscript. XG edited the manuscript, and all authors approved the final manuscript.
